# Fast track to stroke unit for patients not eligible for acute intervention, a case–control register study on 1066 patients

**DOI:** 10.1038/s41598-023-48007-6

**Published:** 2023-11-27

**Authors:** Ingela Wennman, Helle Wijk, Katarina Jood, Eric Carlström, Bengt Fridlund, Linda Alsholm, Johan Herlitz, Per-Olof Hansson

**Affiliations:** 1 Institute of Health and Care Sciences, Sahlgrenska Academy, University of Gothenburg, 405 45 Gothenburg, Sweden; 2 Gothenburg Emergency Medicine Research Group (GEMREG), Sahlgrenska University Hospital, Gothenburg, Region Västra Götaland Sweden; 3 Department of Clinical Neuroscience, Institute of Neuroscience and Physiology, The Sahlgrenska Academy, at the University of Gothenburg, Gothenburg, Sweden; 4 Department of Neurology, Sahlgrenska University Hospital, Region Västra Götaland, Gothenburg, Sweden; 5 Centre for Interprofessional Collaboration Within Emergency Care (CICE), Linnaeus University, Växjö, Sweden; 6 PreHospen – Centre for Prehospital Research, Faculty of Caring Science, Work Life and Social Welfare, University of Borås, Borås, Sweden; 7 Department of Molecular and Clinical Medicine, Institute of Medicine, Sahlgrenska Academy, University of Gothenburg, Gothenburg, Sweden; 8 Department of Medicine, Geriatrics and Emergency Medicine, Region Västra Götaland, Sahlgrenska University Hospital, Gothenburg, Sweden

**Keywords:** Stroke, Health care

## Abstract

Stroke patients not eligible for acute intervention often have low priority and may spend long time at the emergency department (ED) waiting for admission. The aim of this retrospective case–control register study was to evaluate outcomes for such “low priority” stroke patients who were transported via Fast Track directly to the stroke unit, according to pre-specified criteria by emergency medical service (EMS). The outcomes of Fast Track patients, transported directly to stroke unit (cases) were compared with the outcomes of patients who fulfilled these critera for Fast Track, but instead were transported to the ED (controls). In all, 557 cases and 509 controls were identified. The latter spent a mean time of 237 min in the ED before admission. The 90-day mortality rate was 12.9% for cases and 14.7% for controls (n.s.). None of the secondary outcome events differed significantly between the groups: 28-day mortality rate; death rate during hospitalisation; proportion of pneumonias, falls or pressure ulcers; or health-related outcomes according to the EQ-5D-5L questionnaire. These findings indicates that the Fast Track to the stroke unit by an EMS is safe for selected stroke patients and could avoid non-valuable time in the ED.

## Introduction

Stroke is a leading cause of death and disability worldwide^1^. In recent decades, there have been considerable improvements in stroke care, with effective, evidence-based treatments such as intravenous thrombolysis, thrombectomy and care at stroke units^2–4^. Time to acute intervention is a crucial factor in stroke care^2,3^. This knowledge has resulted in an awareness of the importance of early recognition of stroke symptoms, rapid transport to hospital (‘Stroke Alert’) and optimisation of in-hospital stroke care pathways workflow^5–9^. However, most stroke patients are ineligible for acute intervention and are therefore not considered for Stroke Alert^10^. The historical care pathway transfer for these patients is via the emergency department (ED) before admission to the stroke unit. Studies have shown that the ED environment is complex and entails patient safety risks. It has been reported that frequent causes for adverse events in the ED are related to human error^11^, crowding of patients awaiting care^12–14^ and high workloads in combination with a lack of communication skills among staff^11,15,16^. Other negative factors also occur in prehospital settings, such as routines not being fully implemented^17^ and wide variation concerning professionals’ adherence to prehospital and ED guidelines^18^ where the adherence to guidelines is sometimes low^19^.

Because stroke patients are immobile and often elderly, there is a higher risk of complications such as pressure ulcers (PUs)^20^. PUs are a challenge throughout the world and are considered adverse events. The incidence and prevalence are indicators of the quality of care and around the world there are considerable variations observed between different clinical settings and geographical areas^21^. In Sweden, the hospital-acquired PUs rate was 10% (2022)^22^. Another complication is falling, a common and potentially deleterious consequence of stroke^23^.

The patient safety situation is not an isolated problem for the ED, as it is also influenced by what is happening in the preceding part of the care pathway, the EMS system and prolonged waiting time in the ED due to lack of admission to the patient ward^15,24^. This problematic condition means that staff from across the care pathway are involved in protecting patients from adverse events and trying to speed up the process in the care pathway^25^. Leaving the ED environment for interprofessional team care in the stroke unit has proven beneficial. For example, the unit provides early screening for dysphagia and takes preventive action against pneumonia^26^. In addition, patients who have been treated in a stroke unit are likelier to be alive and to be independent at home^4^. Efforts that decrease the number of patients who are referred to the ED and that minimise time spent in the ED decrease patient safety risks and promote rapid transport to the stroke unit; therefore, such efforts are important acute stroke care actions.

In the early 2000s, at a university hospital in western Sweden, a Fast Track was developed for suspected stroke patients who did not meet the criteria for acute intervention. This Fast Track involved patients with a suspicion of stroke being directly transported by an EMS to the stroke unit. The intention was to shorten the time in the care pathway, reduce health-associated harms and use common resources more efficiently. An early evaluation showed that this Fast Track process was associated with relatively high diagnostic accuracy in terms of stroke-related diagnoses and a markedly decreased time delay from initial contact with the 112 to arrival at the stroke unit^27^. However, the safety and potential benefits for the patients needed to be confirmed in a larger study, with a concomitant evaluation of the clinical consequences that also highlighted health-related quality of life (QoL) by using patient reported outcome measurements.

The aim of the present study was thus to describe outcomes related to patient safety for stroke patients considered not eligible for acute intervention but still transported via Fast Track directly to the stroke unit, and to compare these patients´ outcomes with the outcomes of patients considered but not accepted for Fast Track.

## Methods

### Design and study population

This was a register study with a retrospective case–control design. The study population comprised stroke patients at a university hospital in western Sweden serving about 700,000 inhabitants. The university hospital was located on three separate sites in the area, each with its own stroke unit. The EMS included several stations in the area, all publicly run. The ambulances were staffed with at least one Registered Nurse, sometimes with Clinical Nurse Specialist education^28^. The EMS staff had been specially trained in recognising stroke symptoms and patients in whom Stroke Alert was required. According to a checklist, including checkpoints and pre-defined criteria, the EMS nurses contacted the stroke physician on call at the hospital when a Stroke Alert was in effect. In such cases, these patients were referred directly to the computerised tomography lab for an acute brain scan at the hospital.

Since 2008, some patients with a suspected stroke who have not met the criteria for Stroke Alert have been admitted to another Fast Track pathway to the stroke unit according to certain criteria^27^. Patients are considered eligible for this Fast Track when the EMS nurse suspects an acute stroke (acute onset of a neurological deficit) and the patient does not have any pre-defined exclusion criteria according to the checklist. Exclusion criteria were as follows: meeting the criteria stroke alert, signs or symptoms of myocardial ischemia, any convulsion or other epileptic manifestation, plasma-glucose > 22 mmol/L, body temperature > 39.0 °C, oxygen saturation (POX) < 90%, systolic blood pressure < 100 mm Hg, heart rate < 50 or > 110 beats per minute, respiratory rate > 25 breaths per minute or lowered alertness. Minor adjustments have been made to the checklist over the years, but the content has been essentially the same. If an EMS nurse determined that a patient met the Fast Track criteria (patient not eligible for Stroke Alert), a hospital stroke coordinator was contacted by phone. The coordinator double-checked that no exclusion criteria were present and checked for an available hospital bed at any of the stroke units. If the patient was accepted, the EMS transported the patient directly to the stroke unit. The physician on call was notified and examined the patient shortly after the patient’s arrival at the stroke unit. If not accepted for this Fast Track, the patient was transported to the ED, and admission to a hospital ward was managed according to normal routines.

**Figure 1:**
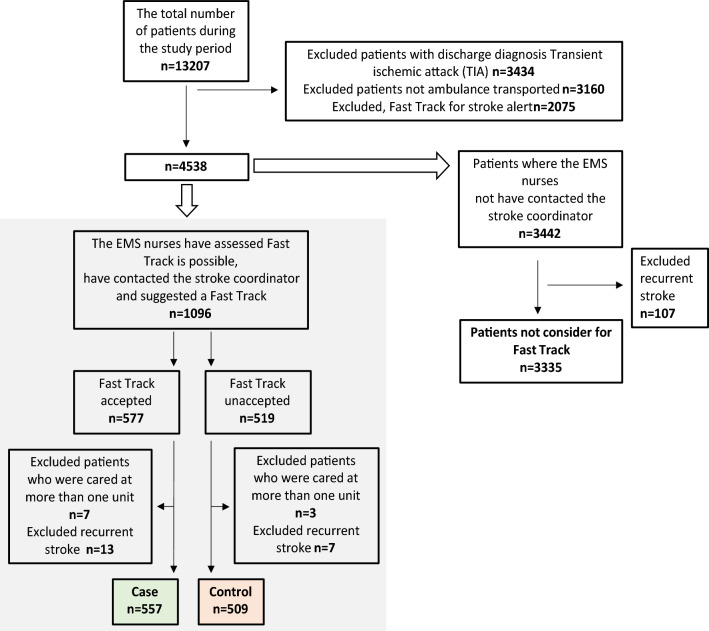
Flowchart of the study enrolment process.

In the present study, the case group consisted of stroke patients transported by an EMS directly to a stroke unit at a university hospital between 1 January 2013 and 31 December 2019. These cases were compared with stroke patients whom, during the same period, the EMS nurse considered eligible for Fast Track—prompting the EMS nurse to contact the stroke coordinator—but who, for some reason, were not accepted for Fast Track and were instead transported to the ED and then later admitted to a stroke unit (controls). In the study population patients were only included if they spent their entire hospital stay at a stroke unit (i.e., they were not treated at any other ward during the hospital stay). Each patient was included only once in the study; thus, recurrent stroke events during the study period were excluded. At two of the stroke units, Fast Track admission was possible 24 h a day and 7 days a week; at the third stroke unit, this strategy was only available Monday through Friday from 8 a.m. to 4 p.m. (Fig. 1).

This study was approved by the Swedish Ethical Authority (Dnr 284-17 and 2021-05163).

Since this is a retrospective register based study, and only observational data were collected, written informed consent are not required according to national law. All methods were performed in accordance with the relevant guidelines and national regulations.

### Data collection

To describe and compare patient safety outcomes in the entire care pathway from the EMS assessment to discharge from the stroke unit, data were collected from several registers. Initially, the study population was identified from the Väststroke register^29,30^, a local quality register established in April 2012 and was completed in December 2019. Prehospital variables were not included at start and was added in 2013. The Väststroke register complements the quality register for stroke at the national level^10,31^ with local data covering the entire care pathway, including prehospital data and outcomes for all patients discharged with a diagnosis of stroke or transient ischemic attack (TIA) from a stroke unit at the university hospital.

In this study, patients treated at the university hospital with a hospital discharge diagnosis of ischemic stroke (ICD-10: I63.0–I63.9) or intracerebral haemorrhagic (ICD 10: I61.0–I61.9) were included. Patients with a discharge diagnosis of unspecified stroke (ICD-10: I64.9) were considered ischemic stroke patients, since all patients were examined with computer tomography of the brain and thereby intracerebral haemorrhagic was identified. Patients with a TIA diagnosis were excluded.

Health-related outcomes according to the European Quality of Life-5 Dimensions on a five-level scale of severity (EQ-5D-5L) were described three months after admission. This instrument has been increasingly applied in populations with various diseases and has been found to have good reliability and sensitivity^32^. In the Väststroke register, the patient-reported data were collected from a questionnaire sent by post. The instrument registers a patient’s health status in five levels of severity, from 1 (no difficulties) to 5 (severe difficulties). The five dimensions evaluated are mobility, hygiene, main activities, pain/discomfort and anxiety/depression. The questionnaire also included an individual self-estimated health condition and a visual analogue scale (VAS) ranging from 0 to 100 (100 = today’s best imaginable health state)^33–35^.

The following data about patients who were accepted and/or considered for Fast Track were obtained from the prehospital and the hospital’s patient administrative data systems: proportion denied due to the number of available hospital beds; total care time in the ED; some secondary discharge diagnoses during hospitalisation, such as pneumonia (ICD-10: J.09-J.22) and confusion (ICD-10: R 41.0, F05); and the assessed triage level within the EMS. The triage system used was the Rapid Emergency Triage and Treatment System (RETTS), which consists of a five-level scale. On this scale, a patient’s status is blue (has no need for ED resources), green (is able to wait), yellow (faces no medical risk from waiting), orange (has a potentially life-threatening condition) or red (has a life-threatening condition)^36^. RETTS for adults in the prehospital setting has the characteristic of detecting a time-sensitive condition but with lower specificity^37^.

Finally, data were also collected on all patients from the National Swedish Stroke Register^10^ about their living situation, smoking habits, comorbidity, medication, mortality and stroke symptoms upon arrival at the stroke unit, according to the National Institutes of Health Stroke Scale (NIHSS). On this scale, which measures several aspects of brain function, a maximum score of 42 represents the most severe stroke, 1–4 represents a minor stroke and 5–15 represents a moderate stroke^38^; this scale has been proven reliable and valid in stroke trials^39^.

### Predefined outcome events

The primary outcome event was death within 90 days after admission. The secondary outcome events were (a) death within 28 days after admission, (b) death during hospitalisation, (c) pneumonia during hospitalisation, (d) fall during hospitalisation, (e) PUs during hospitalisation (f) patient self-reported health-related outcomes according to the EQ-5D-5L three months after admission.

### Statistical analysis

All the data from different registers and hospital systems were merged into one data set and analysed using version 9 of the SAS System for Windows. All significance tests were two-sided and conducted at the 5% significance level.

For categorical variables, number and percentage are presented, while mean with standard deviation (SD) as well as median, minimum and maximum values were presented for continuous variables.

For unadjusted comparisons between the two groups, Fisher’s exact test was used for dichotomous variables, the Mantel–Haenszel chi square test was used for ordered categorical variables, the chi square test was used for non-ordered categorical variables and Fisher’s non-parametric permutation test for comparison of independent means was used for continuous variables.

For analyses of dichotomous outcome variables, univariable and multivariable logistic regressions were performed, and the main results were unadjusted and adjusted odds ratios with 95% confidence intervals (CIs) and p-values.

A sensitivity analysis was performed, for univariable and multivariable analysis for outcome event where all patients with triage colours orange and red were excluded.

Due to the non-randomised design of the study, we had to adjust the analyses for important confounders. Nine pre-defined confounders was identified: age, sex, atrial fibrillation, anticoagulation treatment, EMS nurses’ triage assessment according to RETSS, year, smoking, NIHSS value, type of stroke. Due to many missing values on some baseline variables and a limited number of outcome events, it was not possible to adjust for all known predictors simultaneously. Instead, a baseline confounder was defined as a variable that has a standardized mean difference (SMD) of 0.20 or higher between the two groups and the confounder should also be a significant predictor to the event. Then the results should be adjusted for these confounders.

## Results

**Figure 2:**
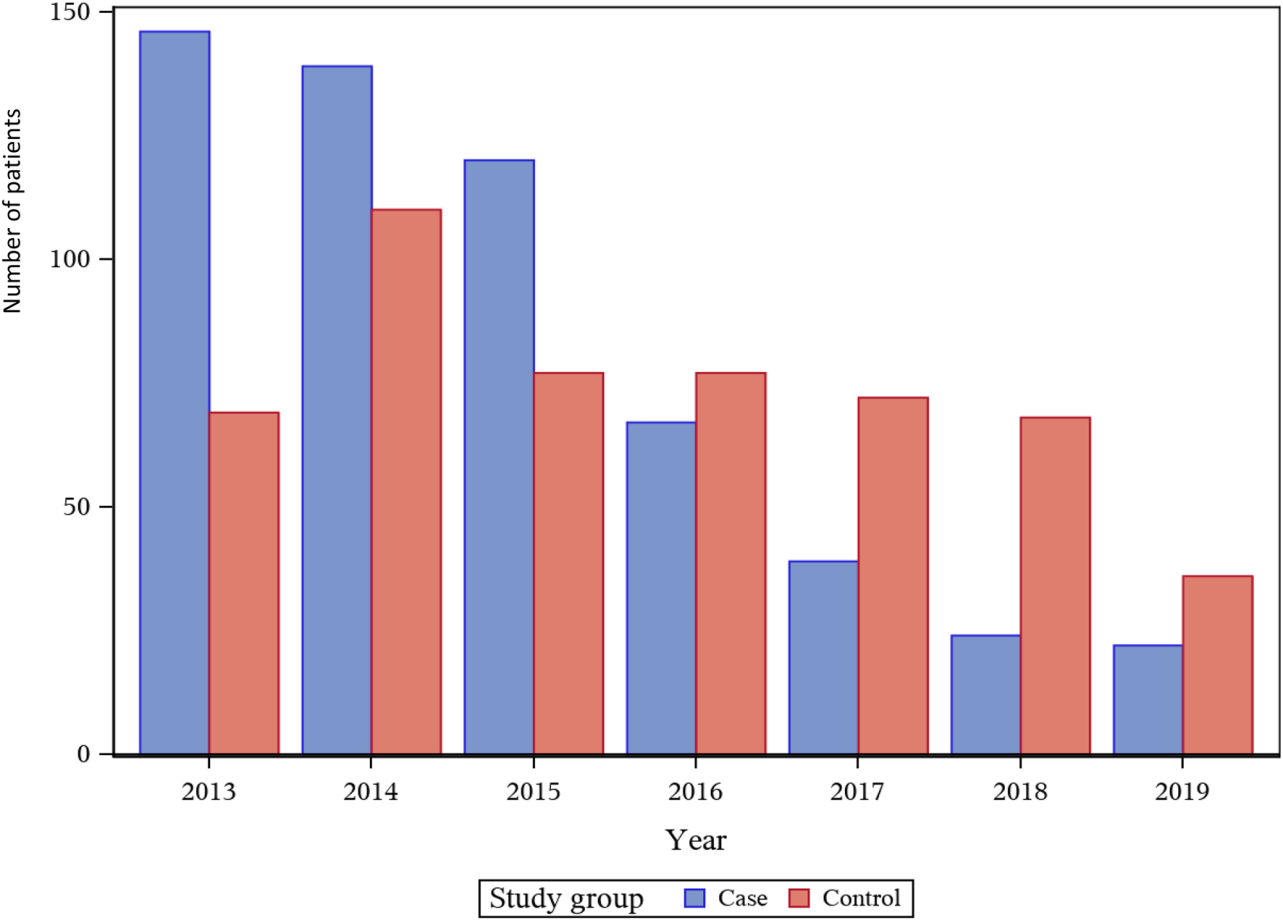
The number of stroke patients admitted directly to stroke unit (case) and stroke patients admitted via the ED (control) per year during the study period.

A total of 1066 patients met the inclusion criteria and were included in the study population. Of these, 557 (52%) were accepted for Fast Track (cases), while 509 were not accepted and were therefore transported to the ED (controls) (Fig. 1). The median age was 81 years, and 50% were women (Table 1). There was a yearly decreasing trend regarding the number of contacts from the EMS nurse to the stroke coordinator; the number decreased from 215 (2013) to 197 (2015) to 111 (2017) to 58 (2019). Additionally, the number of patients accepted for Fast Track decreased over the years, from 68% in 2013 to 38% in 2019. The number of cases and controls per year is shown in Fig. 2. Information about reasons for non-acceptance is not available for most patients. In the prehospital system, it was possible to record the cause ‘no available hospital bed’, which was increasingly frequent over the study period: 8% of patients in 2013 and 45% in 2018 (data not shown).

**Table 1:** Baseline characteristics, cormorbidity and clinical findings comparing 557 stroke patients admitted directly to stroke unit, Fast Track, (cases) and 509 patients not accepted for Fast Track and therefore admitted via the ED (controls).

Characteristics	Total patients	Accepted fast track (cases)	Not accepted fast track (controls)	P-value	Standarized mean difference (SMD) (effect size)
	n = 1066	n = 557	n = 509		
Age, years					
Median (min; max)	81.2 (27; 104)	81.4 (45; 104)	80.4 (27; 99)		
Mean (SD)	79.4 (11.2)	79.9 (10.9)	78.9 (11.5)	0.15	0.088
Sex, n(%)					
Woman	533 (50.0%)	280 (50.3%)	253 (49.7%)	0.90	0.01
Living support n(%)					
* Missing n(%)*	*223 (20.9%)*	*117 (21.0%)*	*106 (20.8%)*		
Living alone without help	514 (61.0%)	271 (61.6%)	243 (60.3%)		
Living alone with help	185 (21.9%)	93 (21.1%)	92 (22.8%)		
Residential care facility	144 (17.1%)	76 (17.3%)	68 (16.9%)	0.87	
Living situation n(%)					
* Missing n(%)*	*228 (21.4%)*	*120 (21.5%)*	*108 (21.2%)*		
Living alone	519 (61.9%)	272 (62.2%)	247 (61.6%)		
Living together	319 (38.1%)	165 (37.8%)	154 (38.4%)	0.85	
Current smoker					
* Missing n(%)*	*335 (31.4%)*	*172 (30.9%)*	*163 (32.0%)*		
Yes	121 (16.6%)	76 (19.7%)	45 (13.0%)	0.018	0.18
Previous stroke n(%)					
Yes	248 (23.3%)	125 (22.4%)	123 (24.2%)	0.51	
Atrial fibrillation n(%)					
Yes	193 (18.1%)	76 (13.6%)	117 (23.0%)	0.0001	0.24
Diabetes mellitus n(%)					
Yes	179 (16.8%)	103 (18.5%)	76 (14.9%)	0.14	
Anticoagulant treatment (on arrival) n(%)					
Yes	71 (6.7%)	12 (2.2%)	59 (11.6%)	< .0001	0.38
Antihypertensiv medication (on arrival) n(%)					
Yes	508 (47.7%)	260 (46.7%)	248 (48.7%)	0.54	
EMS nurses´ triage assessment according to RETTS					
* Missing n(%)*	155 (14.6%)	88 (15.8%)	67 (13.2%)		
Red	25 (2.7%)	12 (2.6%)	13 (2.9%)		
Orange	230 (25.3%)	53 (11.3%)	177 (40.0%)		
Yellow	638 (70.0%)	392 (83.5%)	246 (55.7%)		
Green	18 (2.0%)	12 (2.6%)	6 (1.4%)	< .0001	0.584
Time of day at hospital arrival n(%)					
* Missing n(%)*	*7 (0.7%)*	*5 (0.9%)*	*2 (0.4%)*		
8 AM to 4 PM	670 (63.3%)	329 (59.6%)	341 (67.3%)		
4 PM to 10 PM	270 (25.5%)	167 (30.3%)	103 (20.3%)		
10 PM. to 8 AM	119 (11.2%)	56 (10.1%)	63 (12.4%)	0.0010	
NIHSS, value total					
* Missing n(%)*	574 (53.8%)	324 (58.2%)	250 (49.1%)		
Median (min; max)	3 (0; 36)	3 (0; 30)	3 (0; 36)		
Mean (SD)	4.35 (5.25)	4.15 (5.10)	4.54 (5.38)	0.41	0.074
Type of stroke					
Haemorrage n(%)	58 (5.4%)	27 (4.8%)	31 (6.1%)		0.05
Infarction n(%)	1008 (94.6%)	530 (95.2%)	478 (93.9%)	0.45	0.05
Clinical events during hospitalisation					
Urinary track catheter during hospital stay n(%)	154 (14.4%)	71 (15.3%)	83 (18.2%)	0.27	
Treatment urinary tract infection n(%)	146 (13.7%)	82 (15.1%)	64 (12.7%)	0.31	
Recurrent stroke during hospital stay n(%)	18 (1.7%)	8 (1.5%)	10 (2.0%)	0.69	
Confusion during hospital stay n(%)	4 (0.4%)	2 (0.4%)	2 (0.4%)	1.00	
Length of hospital stay (days)					
Median (min; max)	10.1 (1; 116)	9.8 (1; 80)	10.5 (1; 116)		
Mean (SD)	14.3 (13.6)	13.8 (13.1)	14.9 (14.2)	0.19	

*NIHSS* National Institutes of Health Stroke Scale, *SD* standard deviation.

The controls’ mean time in the ED before transport to the stroke unit was 237 min (median time = 202 min; min. 13 min and max. 1589 min). The mean hospital stay was 13.8 days for the cases and 14.9 days for the controls (p = 0.19) (Table 1).

Baseline data showed that the case group consisted of a significantly higher proportion of patients registered as current smokers (19.7% vs. 13%, p = 0.018). Atrial fibrillation (AF) was more common among controls than among cases (23% vs. 13.6%, p = 0.0001), and a higher proportion of controls were on oral anticoagulation (11.6% vs. 2.2%, p < 0.0001). A higher proportion of controls than cases were assigned orange or red status according to RETTS, and slightly more cases arrived at the hospital in the evening (4 p.m. to 10 p.m.), while there was no difference in median NIHSS value (Table 1).

**Figure 3:**
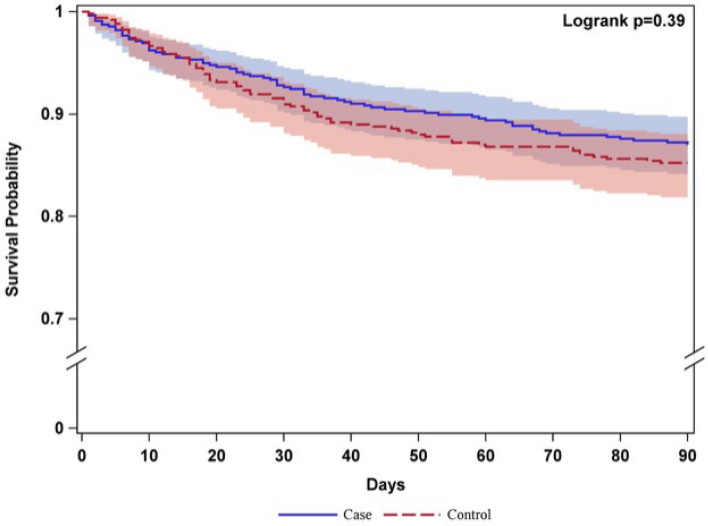
Survival curves up to 90 days after admission, comparing 557 patients admitted directly to stroke unit (cases) and 509 patients admitted via the ED (controls).

The 90-day mortality rate was 12.9% among cases and 14.7% among controls (p = 0.39) (Fig. 3 and Table 2). In the univariable analysis, none of the following outcome events differed significantly between cases and controls: 28-day mortality rate; death during hospitalisation; or proportion of patients with pneumonia, falls or decubitus ulcers (Table 2). Four variables have a standardised mean difference (SMD) > 0.20: AF, anticoagulant treatment and triage colour (Table 1), and year of stroke (p =  < 0.0001, SMD = 0.49). These variables are analysed against our primary and secondary outcomes. Of these baseline variables, only AF was a significant predictor of all three death variables and PUs. This implies that the main analyses were adjusted only for the confounder AF (Table 2).

**Table 2:** Univariable and Multivariable adjusted outcome events comparing 557 patients admitted directly to stroke unit (cases) and 509 patients admitted via the ED (controls).

Outcome variable	Total patients case/control	Accepted fast track (case)	Unaccepted fast track (control)	Univariable		Multivariable	
				OR (95% CI)	P-value	OR (95% CI)	P-value adjusted*
	n = 1066	n = 557	n = 509				
Death within 90 days after admission n (%)	147 (13.8%)	72 (12.9%)	75 (14.7%)	1.16 (0.82–1.65)	0.39	1.06 (0.74–1.51)	0.75
Death within 28 days after admission n (%)	80 (7.5%)	37 (6.6%)	43 (8.4%)	1.29 (0.82–2.04)	0.27	1.19 (0.75–1.88)	0.46
Death during hospitalisation n (%)	68 (6.4%)	35 (6.3%)	33 (6.5%)	1.03 (0.63–1.69)	0.89	0.96 (0.58–1.57)	0.87
Pneumonia during hospitalisation n (%)	24 (2.3%)	13 (2.3%)	11 (2.2%)	0.93 (0.42–2.06)	0.86	0.91 (0.41–2.02)	0.82
Fall during hospitalisation n (%)	101 (9.7%)	52 (9.6%)	49 (9.9%)	1.02 (0.68–1.54)	0.91	0.99 (0.66–1.50)	0.97
Missing n (%)	30 (2.8%)	18 (3.2%)	12 (2.4%)				
Pressure ulcers during hospitalisation n (%)	39 (3.7%)	18 (3.3%)	21 (4.2%)	1.26 (0.67–2.38)	0.47	1.08 (0.57–2.06)	0.81
Missing n (%)	21 (2.0%)	15 (2.7%)	6 (1.2%)				
Self-rated health state on VAS (EQ-5D-5L) (mean (SD))	61.3 (24.2)	62.8 (23.8)	59.8 (24.7)		0.19		0.36
Missing n (%)	631 (59.2%)	333 (59.8%)	298 (58.6%)				

*In multivariable analyses adjusted for atrial fibrillation.

The patients’ self-reported health-related QoL three months after admission to the stroke unit showed no significant difference between the two groups in any of the five dimensions of the EQ-5D-5L. The two groups showed similar patterns for the 5 dimensions. The dimensions relating to hygiene and anxiety/depression were rated the least difficult, while the dimension relating to main activities was rated the most difficult (Table 3). The self-rated health state on the VAS was slightly higher in the case group in mean (62.8) than in the control group (59.8) (p = 0.19) (Table 2).

**Table 3:** Results from EQ-5D-5L questionnaire three months after admission at stroke unit comparing patients admitted directly to stroke unit (cases) and patients admitted via the ED (controls).

Dimensions	Levels	Total (n = 1066)	Case (n = 557)	Control (n = 509)	p-value
EQ1: Mobility n (%)	No difficulties	145 (35.8%)	72 (35.0%)	73 (36.7%)	
		98 (24.2%)	54 (26.2%)	44 (22.1%)	
	Moderate difficulties	76 (18.8%)	41 (19.9%)	35 (17.6%)	
		37 (9.1%)	18 (8.7%)	19 (9.5%)	
	Severe difficulties	49 (12.1%)	21 (10.2%)	28 (14.1%)	0.50
	*Missing data (n)*	661	351	310	
EQ2: Hygiene n (%)	No difficulties	228 (55.9%)	111 (53.6%)	117 (58.2%)	
		68 (16.7%)	38 (18.4%)	30 (14.9%)	
	Moderate difficulties	47 (11.5%)	24 (11.6%)	23 (11.4%)	
		22 (5.4%)	11 (5.3%)	11 (5.5%)	
	Severe difficulties	43 (10.5%)	23 (11.1%)	20 (10.0%)	0.56
	*Missing data (n)*	658	350	308	
EQ3: Main activities n (%)	No difficulties	137 (33.6%)	68 (32.9%)	69 (34.3%)	
		95 (23.3%)	47 (22.7%)	48 (23.9%)	
	Moderate difficulties	67 (16.4%)	38 (18.4%)	29 (14.4%)	
		45 (11.0%)	19 (9.2%)	26 (12.9%)	
	Severe difficulties	64 (15.7%)	35 (16.9%)	29 (14.4%)	0.71
	*Missing data (n)*	658	350	308	
EQ4: Pain or discomfort n (%)	No difficulties	155 (38.5%)	84 (40.6%)	71 (36.2%)	
		90 (22.3%)	39 (18.8%)	51 (26.0%)	
	Moderate difficulties	108 (26.8%)	59 (28.5%)	49 (25.0%)	
		43 (10.7%)	23 (11.1%)	20 (10.2%)	
	Severe difficulties	7 (1.7%)	2 (1.0%)	5 (2.6%)	0.73
	*Missing data (n)*	663	350	313	
EQ5: Anxiety or depression n (%)	No difficulties	156 (38.3%)	87 (42.0%)	69 (34.5%)	
		163 (40.0%)	74 (35.7%)	89 (44.5%)	
	Moderate difficulties	54 (13.3%)	28 (13.5%)	26 (13.0%)	
		27 (6.6%)	16 (7.7%)	11 (5.5%)	
	Severe difficulties	7 (1.7%)	2 (1.0%)	5 (2.5%)	0.46
	*Missing datal (n)*	659	350	309	

The sensitivity analysis, which excluded all cases and controls with triage colours orange or red from the analysis, showed similar results for all outcome events, and there was no difference between cases and controls (data not shown).

## Discussion

In the present study, the aim was to describe outcomes related to patient safety for stroke patients considered not eligible for acute intervention but still transported via Fast Track directly to the stroke unit, and to compare these patients´ outcomes with the outcomes of patients considered but not accepted for Fast Track. The results showed no significant differences between the two groups regarding patient safety-related outcomes. (Complications in the acute phase or health-related outcomes three months after admission to the stroke unit). From 2013 to 2019, the number of denied Fast Tracks increased. The main reason for not being accepted was the lack of available patient beds at the stroke unit, which may explain why slightly more patients were accepted for Fast Track in the evening (4 p.m. to 10 p.m.) since discharges of inpatients often take place later in the day, which frees up hospital beds. We have previously reported that when EMS staff frequently met with the message ‘unaccepted Fast Track’, they described reluctance and ‘a feeling of giving up’, which resulted in their not even trying to initiate Fast Tracks^24^. This is likely one reason for the decreasing number of contacts from the EMS to hospital stroke coordinators, from 215 (2013) to 58 (2019). Other likely reasons are improvements in treatment options and increased time window for acute interventions^2,3,40,41^, resulting in more patients being eligible for acute intervention and therefore transported via another Fast Track process, such as Stroke Alert, directly to computer tomography and consideration for reperfusion treatment^10,29^.

Studies have indicated that professionals’ adherence to prehospital and ED guidelines varies greatly^18^ and that compliance with guidelines is sometimes low in the prehospital setting^19^. Some of the patients whom the EMS nurses tried to involve in the Fast Track did not match the predefined criteria. There are probably several causes for that, but one could be that EMS nurses by phone consulted the stroke-physician who accepted direct admission, even though all criteria were not met. Wennman et al. (2022) found that staff throughout the entire care pathway cooperated in flexible ways, sometimes outside routines, in order to protect patients from safety risks, ED crowding and delays in the care pathway^25^.

Previous studies have pointed out (a) that the ED environment entails patient safety risks^11–16^ and (b) the benefits of leaving the ED environment for interprofessional team care in the stroke unit^4,26^. Furthermore, studies have shown that there is a risk of complications, especially for stroke patients, such as pneumonia^42^, falls^23^ and Pus—a complication that often occurs in hospitals^20^. Therefore, this study’s result –showing no significant difference between the groups in any of the predefined outcome events—was unexpected. However, since the present study population consisted of a selected group of stroke patients with relatively low severity, it is difficult to compare these results with general data describing the entire population.

In the present study, there was a low frequency of post-stroke pneumonia (2.3%). A systematic review by Badve et al. (2018) concluded that 10% of stroke patients experience pneumonia during the acute period of hospital care. Furthermore, the incidence of falls is reported to be 13%–22% during hospitalisation^23^, compared to 9.7% in the present study. The prevalence of PUs (3.7%) was also lower in the present study than in the overall data in Sweden (the hospital-acquired PUs rate, 10%, 2022)^22^. The fact that the frequency of pneumonia, falls and PUs was lower in this study than in others could be explained by the high quality of the stroke care, the selection of ‘low risk’ stroke patients or both.

Although this study did not show any significant differences between the groups, the controls’ mean time spent in the ED before transport to the stroke unit was long: 237 min. Prehospital data showed that the main cause of transport to the ED was a lack of availability of hospital beds. This situation is a long-term trend (2000–2018) across Europe. OECD data show that the number of hospital beds per capita is declining^43^. Although this situation can be partly explained by increased possibilities in diagnostics and clinical care leading to more efficient use of hospital resources, Sweden stands out regarding the number of hospital beds. Among OECD-countries in 2000, Sweden had almost the lowest number of hospital beds per 1000 population (about 3.7), and in 2018, it did have the lowest number (about 2.1)^43^; moreover, in Sweden the mean time spent at the ED tends to be increasing, according to national statistics^44^. It is known, except for suddenly suffering from a serious illness and be in a critical health related transition^45^, long time spent at the ED is associated with low patient satisfaction^46^. To improve patient turnover and release hospital beds, feedback systems between hospitals and bed capacity control systems has proven to be a remedy^47^. However, in Beveridge-like health care systems like the one in Sweden, such remedies are either rare or insufficient^48^. In Sweden a slow hospital throughput is known to be an effect of staff shortage, and poor incentives to improve the bed capacity. The standardized and fixed salaries in the Swedish tax financed and public health may contribute to bed-blocking and a minimized patient turnover^49^. More hospital beds and improved personal incentives among nurses and clinicians may pave the way for solutions to the crowding issue. On the other hand, patient experience is positively associated with both patient safety and clinical effectiveness^50^. In Swedish patient law, patient safety refers to health-associated harm: ‘suffering bodily or psychological injury or illness and deaths, that could have been avoided if sufficient measures had been taken in the patient’s contact with the healthcare system’^51^.

Therefore, patient safety in the Fast Track process from the EMS to the stroke unit should be studied in light of both psychological and bodily suffering.

### Strengths and limitations

The strengths of the study were the relatively large sample size and the fact that the study population was recruited from a well-defined area with a limited number of hospital departments and EDs. Additionally, data were merged from several high-quality registers, which made it possible to retrieve both objective outcomes and subjective, patient-reported outcomes.

The major weaknesses are the retrospective design and the fact that the study population was not randomised into two groups. The study population was based on the EMS-nurses’ ability to assess stroke symptoms, willingness to contact the hospital coordinator and adherence to guidelines, which could affect representatively. Another weakness could be that the data were aggregated and included three stroke units. Data were collected from several parts of the care pathway, each with its own inherent risk of missing data. The multiplicity of documenting systems is probably one reason for the relatively high proportion of missing values for some variables.

## Conclusion

No difference was detected in predefined patient safety outcomes between stroke patients who spent a mean time of almost 4 h in the ED before being referred to the stroke unit and patients who were admitted directly to the stroke unit. This indicates that the Fast Track to the stroke unit by an EMS is safe for selected stroke patients and could avoid non-valuable time in the ED. A large-scale randomised study could further strengthen this conclusion.

## Data Availability

The datasets used and/or analysis from the study are available from the corresponding author on reasonable request.
